# Insulin Stimulates Adipogenesis through the Akt-TSC2-mTORC1 Pathway

**DOI:** 10.1371/journal.pone.0006189

**Published:** 2009-07-10

**Authors:** Hui H. Zhang, Jingxiang Huang, Katrin Düvel, Bernard Boback, Shulin Wu, Rachel M. Squillace, Chin-Lee Wu, Brendan D. Manning

**Affiliations:** 1 Department of Genetics and Complex Diseases, Harvard School of Public Health, Boston, Massachusetts, United States of America; 2 Urologic Research Laboratory, Massachusetts General Hospital, Department of Pathology, Harvard Medical School, Boston, Massachusetts, United States of America; 3 Biology Department, ARIAD Pharmaceuticals, Inc., Cambridge, Massachusetts, United States of America; Roswell Park Cancer Institute, United States of America

## Abstract

**Background:**

The signaling pathways imposing hormonal control over adipocyte differentiation are poorly understood. While insulin and Akt signaling have been found previously to be essential for adipogenesis, the relative importance of their many downstream branches have not been defined. One direct substrate that is inhibited by Akt-mediated phosphorylation is the tuberous sclerosis complex 2 (TSC2) protein, which associates with TSC1 and acts as a critical negative regulator of the mammalian target of rapamycin (mTOR) complex 1 (mTORC1). Loss of function of the TSC1-TSC2 complex results in constitutive mTORC1 signaling and, through mTORC1-dependent feedback mechanisms and loss of mTORC2 activity, leads to a concomitant block of Akt signaling to its other downstream targets.

**Methodology/Principal Findings:**

We find that, despite severe insulin resistance and the absence of Akt signaling, TSC2-deficient mouse embryo fibroblasts and 3T3-L1 pre-adipocytes display enhanced adipocyte differentiation that is dependent on the elevated mTORC1 activity in these cells. Activation of mTORC1 causes a robust increase in the mRNA and protein expression of peroxisome proliferator-activated receptor gamma (PPARγ), which is the master transcriptional regulator of adipocyte differentiation. In examining the requirements for different Akt-mediated phosphorylation sites on TSC2, we find that only TSC2 mutants lacking all five previously identified Akt sites fully block insulin-stimulated mTORC1 signaling in reconstituted *Tsc2* null cells, and this mutant also inhibits adipogenesis. Finally, renal angiomyolipomas from patients with tuberous sclerosis complex contain both adipose and smooth muscle-like components with activated mTORC1 signaling and elevated PPARγ expression.

**Conclusions/Significance:**

This study demonstrates that activation of mTORC1 signaling is a critical step in adipocyte differentiation and identifies TSC2 as a primary target of Akt driving this process. Therefore, the TSC1-TSC2 complex regulates the differentiation of mesenchymal cell lineages, at least in part, through its control of mTORC1 activity and PPARγ expression.

## Introduction

The ser/thr kinase Akt (or protein kinase B/PKB) plays an essential role in adipocyte differentiation. Mouse embryonic fibroblasts (MEFs) lacking Akt1 (PKBα) display an inability to differentiate into adipocytes [1]–[3], and an RNAi-mediated decrease in Akt1 was found to block the differentiation of 3T3-L1 cells [4], a well-established preadipocyte cell line. Furthermore, constitutively active Akt can promote the differentiation of 3T3-L1 cells into adipocytes, even in the absence of other inputs [5]. While it is clear from these studies that Akt is both necessary and sufficient to drive adipogenesis, its downstream targets involved in regulating this differentiation program are not well understood.

Akt phosphorylates and regulates a large number of substrates involved in a diverse array of biological processes [6], many of which could contribute to the role of Akt in driving adipocyte differentiation. Ultimately, the differentiation defect in MEFs and 3T3-L1 preadipocytes lacking Akt1 stems from an inability to induce peroxisome proliferator-activated receptor γ (PPARγ) expression at the initiation of the adipogenesis program [1], [2], [4]. The PPARγ transcription factor is the master regulator of adipocyte differentiation, and like Akt1, its activation is both necessary and sufficient for differentiation [7]. Supporting an essential role for PPARγ induction downstream of Akt1, forced expression of PPARγ in Akt-deficient MEFs was found to rescue their severe adipogenesis defect [1].

At least two downstream branches of Akt signaling have been implicated in the regulation of PPARγ expression and adipocyte differentiation. The forkhead box O (FOXO) family of transcription factors are phosphorylated and inhibited by Akt. Amongst FOXO family members, FOXO1 expression is highest in adipose tissue, but FOXO1 appears to be a strong inhibitor of adipocyte differentiation [8]. An active mutant of FOXO1 that cannot be phosphorylated and inhibited by Akt was found to block the differentiation of preadipocytes, and this correlated with a loss of PPARγ induction [8]. Furthermore, FOXO1 has been proposed to directly repress PPARγ transcription [9]. Taken together, these studies suggest that Akt-mediated inhibition of FOXO1 is one mechanism by which Akt induces PPARγ and subsequent adipocyte differentiation.

Another major signaling branch downstream of Akt results in activation of the mammalian target of rapamycin (mTOR), a critical regulator of mRNA translation and cell growth [10]. Primarily through use of the mTOR-specific inhibitor rapamycin, several studies have concluded that mTOR activity is required for proper differentiation of preadipocyte cell lines and primary cultures [11]–[16]. The effects of rapamycin were found to correlate with a reduction in PPARγ mRNA and protein levels [12]–[15]. Transcriptional reporter assays in HEK293 cells suggested that rapamycin might also inhibit insulin-stimulated PPARγ activity [15]. Interestingly, mouse knockouts affecting specific mTORC1 targets suggest that both S6K1 activation and 4E-BP1/2 inhibition might contribute to a pro-adipogenic role for mTORC1 activation [17], [18].

The signaling relationship between Akt and mTOR is quite complex [19], [20]. Akt activity results in downstream activation of mTOR complex 1 (mTORC1), which is acutely sensitive to rapamycin. Akt has been proposed to activate mTORC1 through multiple mechanisms. The first is through direct multi-site phosphorylation and inhibition of TSC2 [21], [22]. TSC2, when complexed with its binding partner TSC1, acts as a GTPase activating protein (GAP) for the Ras-related small G protein Rheb, which is a potent activator of mTORC1 [23]–[28]. Therefore, Akt-mediated phosphorylation of TSC2 relieves its ability to act as a GAP for Rheb within cells, allowing Rheb-GTP to accumulate and activate mTORC1. A parallel mechanism appears to be through phosphorylation of the Akt substrate PRAS40 [29]. PRAS40 has been found by several groups to associate with mTORC1, either as a regulatory component or downstream substrate [30]–[36]. Two studies have demonstrated that the previously characterized Akt phosphorylation site on PRAS40 is involved in mTORC1 activation by Akt [30], [31]. Finally, a more indirect and less acute effect of Akt activation on mTORC1 has been proposed to be through the effects of Akt on glucose uptake, intracellular energy levels, and AMPK activity [37]. Importantly, while mTORC1 is clearly downstream of Akt, its activation can lead to feedback inhibition of insulin signaling to Akt through effects on the insulin-receptor substrate (IRS) proteins (reviewed in ref. [38]). Therefore, as originally characterized in 3T3-L1 cells [39]–[43], short-term rapamycin treatment can increase Akt activation in response to insulin in adipocytes and many other cell types.

A second mTOR-containing protein complex, mTORC2, lies upstream of Akt and contributes to Akt activation by insulin and growth factors by phosphorylating S473 in its hydrophobic regulatory motif [44]. mTORC2 is resistant to the acute effects of rapamycin, but prolonged treatment with rapamycin disrupts mTORC2 and in some lines results in loss of Akt phosphorylation [45]. To this end, prolonged rapamycin treatment has been found to block Akt signaling in differentiating 3T3-L1 adipocytes [12], suggesting that some of the previous findings regarding the effects of rapamycin treatment on adipocyte differentiation could be through loss of mTORC2-mediated Akt activation, rather than inhibition of mTORC1. However, a recent study using siRNAs to the mTORC1-specific component Raptor have supported a role for mTORC1 in adipogenesis [46].

*TSC1* and *TSC2* are tumor suppressor genes that are mutated in the autosomal dominant tumor syndrome tuberous sclerosis complex (TSC), which is characterized by widespread benign tumors and a high incidence of epilepsy, autism spectrum disorders, and cognitive deficits (reviewed in ref. [47]). *TSC2* mutations are also the underlying cause of lymphangioleiomyomatosis (LAM [48]), a proliferative disorder that almost exclusively affects women and results in cystic destruction of the lungs. Due to loss of function of the TSC1-TSC2 complex and its GAP activity toward Rheb, lesions from TSC and LAM patients display constitutively elevated mTORC1 signaling. Interestingly, renal angiomyolipomas (AMLs), which are unusual highly vascular tumors comprised of both aberrant smooth muscle cells and adipocytes, are amongst the most common clinical manifestation of the TSC and LAM diseases. Within AMLs, both the smooth muscle and adipose components display loss of heterozygosity (LOH) at the *TSC2* locus [49], demonstrating that both cell types are constituents of the tumor rather than stromal derivatives. This pathological observation might be linked to a role for mTORC1 in driving mesenchymal cell differentiation.

In this study, we take advantage of the signaling changes in cell lines lacking the TSC1-TSC2 complex to determine whether mTORC1 signaling downstream of Akt plays an important role in adipogenesis. Previous studies have demonstrated that MEFs derived from *Tsc1* or *Tsc2* knockout embryos display constitutive activation of mTORC1 [50], [51], strong mTORC1-dependent feedback inhibition of insulin signaling to Akt [50], [52]–[54], and loss of mTORC2 activity [55]. Therefore, disruption of the TSC1-TSC2 complex results in Akt-independent activation of mTORC1 with concomitant loss of Akt signaling to its other downstream targets, including the FOXO proteins [54], [56], [57]. We find that, despite complete loss of Akt signaling, *Tsc1^−/−^* and *Tsc2^−/−^* MEFs display enhanced adipogenic potential, yielding insulin resistant adipocytes containing increased levels of triglycerides. A similar effect is seen in 3T3-L1 cells following stable shRNA-mediated knockdown of *Tsc2*. Importantly, PPARγ mRNA and protein levels are increased in an mTORC1-dependent manner in adipocytes derived from *Tsc2*-deficient MEFs and 3T3-L1 cells. We find that reconstituting *Tsc2^−/−^* MEFs with a mutant of TSC2 lacking all Akt phosphorylation sites blocks insulin-stimulated mTORC1 signaling and greatly reduces adipogenesis. Finally, we find that both the aberrant smooth muscle and adipose components of AMLs from TSC patients exhibit mTORC1 activation and elevated PPARγ expression.

## Materials and Methods

### Cell culture and constructs

Undifferentiated MEFs were cultured in Dulbecco's Modified Eagle's medium (DMEM) with 10% fetal bovine serum. Immortalized littermate-derived pairs of wild-type and null *Tsc1* (3T3 immortalized) and *Tsc2* (p53^−/−^) MEFs were provided by D.J. Kwiatkowski (Brigham and Women's Hospital, Boston, MA) and were described previously [51], [52]. Phosphorylation site (Ser/Thr to Ala) mutants of human TSC2 were generated using QuickChange Multisite-directed Mutagenesis (Stratagene), sequence verified, and subcloned into a previously described retroviral vector (IRES-Hygro, gift of D.J. Kwiatkowski) upstream of an internal ribosome entry sequence followed by the hygromycin-resistance selectable marker [55]. Stable isogenic pools of *Tsc2^−/−^* MEFs expressing the various constructs were generated by infection with retroviruses encoding empty vector, wild-type TSC2, or the TSC2 phosphorylation-site mutants, followed by selection with hygromycin (100 µg/ml). 3T3-L1 preadipocytes were obtained from the American Type Culture Collection and maintained in DMEM with 10% calf serum prior to differentiation.

Stable shRNA-mediated knockdown of *Tsc2* expression in 3T3-L1 preadipocytes was achieved using retroviral contructs. shRNA constructs targeting firefly luciferase or murine *Tsc2* (V2MM_5415) were obtained from Open Biosystems, and the shRNAs were subcloned into the mir30-based pMSCV-PM retroviral vector provided by the laboratory of S.J. Elledge (Harvard Medical School, Boston, MA). Retroviruses encoding these shRNAs were produced in HEK-293T cells and were used to infect 3T3-L1 preadipocytes. Stable shRNA-expressing pools of 3T3-L1 cells were selected with puromycin (2 µg/ml) and were used immediately for the differentiation experiments described.

See Text S1 for supplemental materials and methods.

### Adipocyte differentiation and triglyceride measurements

Previously established protocols for MEF [58] and 3T3-L1 [59] adipocyte differentiation were followed and are described in brief below. For induction of MEF differentiation, at 2 days post confluence (day 0), cells were exposed to a pro-differentiation regimen of insulin (830 nM), dexamethasone (1 µM), 3-isobutyl-1-methylxanthine (IBMX, 0.5 mM), and troglitazone (5 µM). After 48 hours, cells were switched to maintenance DMEM medium containing 10% FBS and 830-nM insulin for the remaining duration of differentiation. Adipocyte differentiation parameters were measured at 7–8 days following the initiation of differentiation. For induction of 3T3-L1 cell differentiation, preadipocytes were grown to 2 days post-confluence and the medium was changed to DMEM supplemented with 10% FBS, insulin (1.7 µM), dexamethasone (1 µM), and IBMX (0.5 mM). After 48 h, the medium was replaced with maintenance medium containing DMEM supplemented with 10% FBS. The maintenance medium was changed every 48 h until the cells were utilized for experimentation (9 days from the initiation of differentiation).

Oil Red O staining and measurement of intracellular triglyceride levels were performed as described by others [60]. For quantification, cells were washed extensively with water to remove unbound dye, and 1-ml isopropanol was added to the stained 6-well culture plate. After 5 min, the absorbance at 510 nm of the Oil Red O extract and serially diluted Oil Red O standards were assayed by spectrophotometer. Triglyceride levels were normalized to protein concentration.

### mRNA expression analysis

For gene expression analyses, RNA was isolated using the RNeasy Mini Kit (Qiagen), cDNAs were generated using the Superscript III First Strand Synthesis System for RT-PCR kit (Invitrogen), and SYBR green-based quantitative RT-PCR was performed using an Applied Biosystems 7300 Real Time PCR System. Samples were run in triplicate for each experiment and were normalized to either β-actin or m36b4 expression to determine relative expression levels. Primer sets used in this study are provided in supplemental Table S1.

### Immunoblotting and immunohistochemistry

Protein extracts/cell lysates and immunoblots were prepared as described previously [22]. All lysates were normalized for total protein concentration prior to SDS-PAGE and immunoblotting. With the exception of the β-actin antibody (Sigma), all antibodies used for immunoblotting were obtained from Cell Signaling Technology.

Formalin-fixed, paraffin-embedded renal AML tissue from TSC patients, and normal human adipose tissue, were obtained from the Pathology Department of Massachusetts General Hospital. This study was approved by the Hospital Human Study Committees. Serial 5-µm tissue sections were cut from each tissue block for immunohistochemical analyses using the avidin-biotin complex (ABC) method. Briefly, slides were deparaffinized and endogenous peroxidase activity was blocked by incubation in 3% H_2_O_2_ for 10 min at room temperature. For antigen retrieval, sections were heated in a pressure cooker in 10-mM citrate buffer (pH 6.0) prior to staining. Antibodies to phospho-S6 (Ser235/236; Cell Signaling Technology #2211; 1∶200 dilution) and PPARγ (Santa Cruz Biotechnology #7196; 1∶100 dilution) diluted in phosphate-buffered saline (PBS) were applied and incubated overnight at 4°C. Negative controls included adjacent sections incubated in PBS without primary antibody. Immunodetection was performed with an LSAB 2 system (DAKO). Hematoxylin was used as a counterstain, and an adjacent section was stained with hematoxylin and eosin.

### Statistical Analysis

Results are expressed as the mean±SEM. Quantitative data for intracellular triglyceride levels were analyzed by one-way analysis of variance or paired 2-tailed Student *t* test using GraphPad InStat version 2.04a. Multiple comparisons were performed using Tukey's honestly significant difference procedure.

## Results

### MEFs lacking the TSC1-TSC2 complex display enhanced mTORC1-dependent adipogenesis

To determine if mTORC1 activation downstream of Akt is both necessary and sufficient to drive adipocyte differentiation, we utilized littermate pairs of wild-type and either *Tsc1^−/−^* or *Tsc2^−/−^* MEFs. Akt and mTORC1 signaling in these MEFs have been characterized in previous studies [50]–[57]. These MEFs display growth factor and Akt-independent activation of mTORC1 signaling, as indicated by phosphorylation of the ribosomal S6 protein, and complete loss of insulin-stimulated Akt phosphorylation (Figure S1, panels A and B). The defect in Akt activation and signaling to its downstream substrates is also evident in TSC2-deficient MEFs grown in full serum, as illustrated by a loss of FOXO1 phosphorylation on T24, and constitutive localization of a FOXO3a-GFP fusion protein to the nucleus in *Tsc2^−/−^* cells, similar to a mutant lacking the three Akt phosphorylation sites (FOXO3a-AAA, Figure S1, panels C and D). Therefore, these cells offer a unique setting in which mTORC1 activation can be completely separated from the many other downstream branches of Akt signaling.

**Figure 1:**
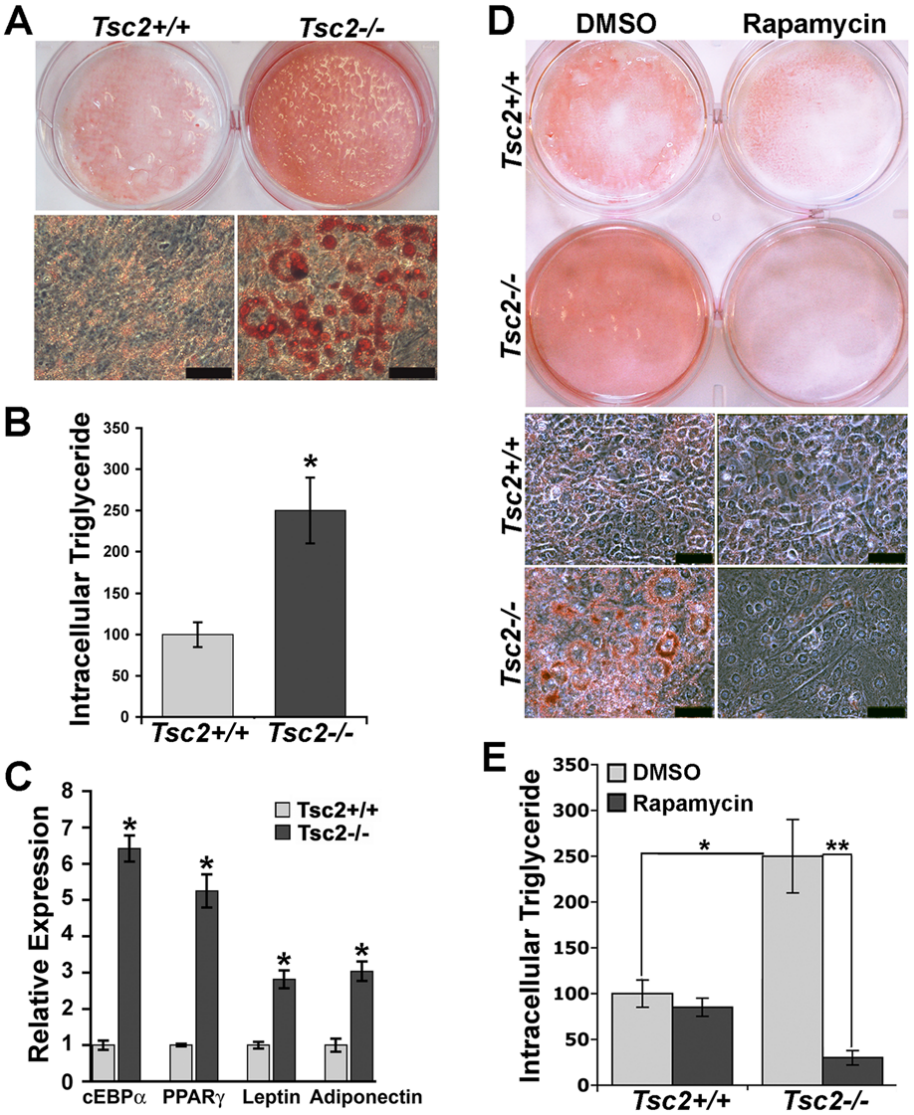
*Tsc2*-deficient MEFs display an mTORC1-dependent increase in adipogenesis. (A) Greater lipid accumulation following an adipocyte differentiation protocol in *Tsc2^−/−^* MEFs relative to littermate-derived *Tsc2^+/+^* MEFs. MEFs of the indicated genotype were induced to differentiate for 8 days, and cells were then stained with Oil Red O. Representative culture dish wells (top) and microscopic fields of view (bottom; scale bars = 25 µm) are shown at the same magnification for each genotype. (B) *Tsc2^−/−^* adipocytes have higher levels of intracellular triglyceride than *Tsc2^+/+^* adipocytes. Relative triglyceride levels, normalized to cellular protein content, are shown from three different experiments as the mean±SEM. *P<0.01. (C) Adipogenic factors and adipocyte markers are elevated in *Tsc2^−/−^* adipocytes. Relative expression of the indicated genes was determined by quantitative RT-PCR and is presented as mean±SEM relative to *Tsc2* ^+/+^ adipocytes (day 8 of differentiation). *P<0.01. (D) Rapamycin inhibits adipocyte differentiation of *Tsc2* ^−/−^ cells. *Tsc2* ^+/+^ and *Tsc2* ^−/−^ MEFs were induced to differentiate for 8 days in the presence of rapamycin (20 nM) or vehicle control (0.1% DMSO), and cells were stained with Oil Red O. Representative culture dish wells (left) and microscopic fields of view (right; scale bars = 25 µm) are shown at the same magnification for each genotype. (E) The increased intracellular triglyceride levels in *Tsc2^−/−^* adipocytes is dependent on mTORC1 activation. *Tsc2* ^+/+^ and *Tsc2* ^−/−^ MEFs were treated as in (D), and relative triglyceride levels, normalized to cellular protein content, were determined and are shown from three different experiments as the mean±SEM. *P<0.01, **P<0.001.

These MEF pairs were subjected to conditions previously established to promote differentiation of MEFs into adipocytes [58]. Importantly, even in response to the very high insulin levels utilized in this differentiation protocol (830 nM), Akt activation is defective in both *Tsc1^−/−^* and *Tsc2^−/−^* MEFs (Figure S1, panels A and B). Interestingly, despite severe insulin resistance, both knockout cell lines underwent morphological changes consistent with adipocyte differentiation and showed a greatly enhanced capacity to generate lipid droplets, as indicated by oil red O staining (Figure 1A and Figure S2A) and intracellular triglyceride levels (Figure 1B and Figure S2B), relative to their wild-type counterparts. To determine whether the increase in lipid accumulation in the *Tsc2*-deficient cells reflects an increase in adipogenesis, at day 7 of differentiation we measured the expression of adipocyte markers, including the adipogenic transcription factors C/EBPα and PPARγ and the adipocyte-specfic cytokines leptin and adiponectin. While transcript levels for all of these markers were induced in both *Tsc2^+/+^* and *Tsc2^−/−^* MEFs over the course of differentiation, their levels were significantly higher in the *Tsc2^−/−^* cells (Figure 1C). Therefore, cells lacking a functional TSC1-TSC2 complex display an enhanced capacity to differentiate into adipocytes.

Given the previous studies demonstrating that rapamycin could block adipocyte differentiation [11]–[16], we hypothesized that the enhanced adipogenesis phenotype of *Tsc2^−/−^* MEFs would be dependent on the elevated mTORC1 activity in these cells. Indeed, *Tsc2^−/−^* MEFs failed to differentiate in the presence of rapamycin, exhibiting a loss of oil red O-staining (Figure 1D) and a dramatic decrease in intracellular triglycerides (Figure 1E). This effect of rapamycin is also seen in differentiating *Tsc1^−/−^* MEFs (Figure S2B). As Akt and mTORC2 activity are already inhibited in cells lacking the TSC1-TSC2 complex [55], these findings demonstrate that the effects of rapamycin on adipocyte differentiation are specific to mTORC1.

### TSC2-deficient MEFs differentiate in an insulin-independent manner and give rise to insulin resistant adipocytes

**Figure 2:**
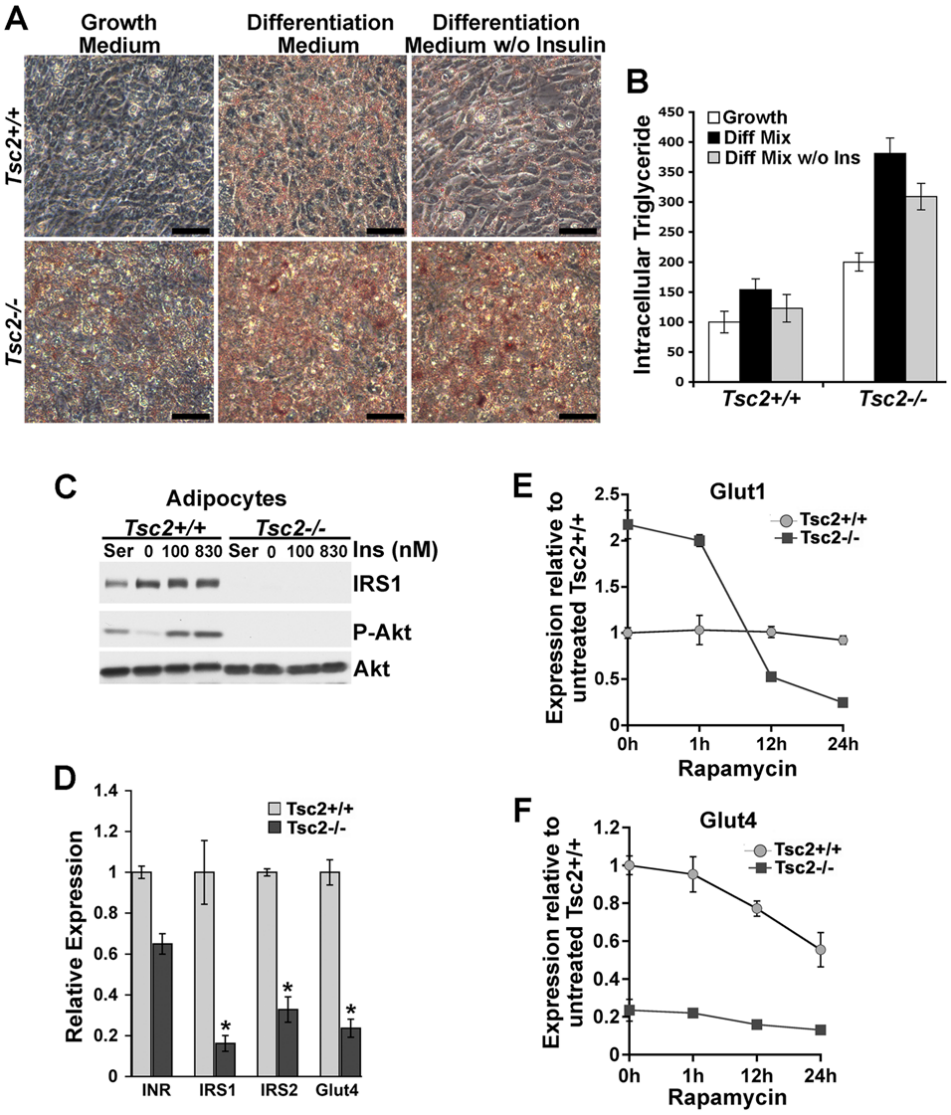
TSC2-deficient MEFs differentiate in an insulin-independent manner and give rise to insulin resistant adipocytes. (A) Insulin-independent differentiation of *Tsc2^−/−^* MEFs. MEFs of the indicated genotype were grown to confluence and then either maintained in normal growth medium or induced to differentiate (see Materials and Methods) in the presence or absence of exogenous insulin (830 nM) for 8 days. Cells were then stained with Oil Red O. Representative fields of cells are shown (scale bars = 50 µM). (B) MEFs were treated as in (A) and relative intracellular triglyceride levels, normalized to cellular protein content, are shown from three different experiments as the mean±SEM. (C) Decreased IRS-1 and insulin resistant Akt activation in differentiated *Tsc2* ^−/−^ adipocytes. Differentiated *Tsc2* ^+/+^ and *Tsc2* ^−/−^ adipocytes (day 8) were serum starved for 16 h and then stimulated with 100- or 830-nM insulin for 15 min prior to lysis and immunoblotting with the indicated antibodies (P-Akt-S473). (D) *Tsc2^−/−^* adipocytes express significantly lower levels of *Irs1*, *Irs2*, and *Glut4* mRNAs. Relative expression of the indicated genes was determined by quantitative RT-PCR and is presented as mean±SEM relative to *Tsc2* ^+/+^ adipocytes (day 8 of differentiation). *P<0.001. (E) *Glut1* transcript levels are elevated in an mTORC1-dependent manner in *Tsc2^−/−^* adipocytes. Differentiated *Tsc2* ^+/+^ and *Tsc2* ^−/−^ adipocytes were treated with rapamycin (20 nM) for 1 h, 12 h, or 24 h prior to determining the expression levels of *Glut1* by quantitative RT-PCR. Expression levels are presented as mean±SEM relative to untreated (0 h) *Tsc2* ^+/+^ cells. (F) Glut4 transcript levels were also measured in the samples described in (C).

Insulin signaling through IRS-1 and IRS-2 has been shown to be an essential element of the adipocyte differentiation program [61]. Given the down-regulation of these proteins in cells lacking the TSC1-TSC2 complex [53], [54], [62], we hypothesized that constitutive mTORC1 activation would allow these cells to differentiate independent of insulin signaling. Interestingly, even in the complete absence of differentiation medium, *Tsc2^−/−^* MEFs incubated at confluence for 7 days in normal growth medium accumulated more lipid than differentiated *Tsc2^+/+^* cells (Figure 2, panels A and B). However, the full adipocyte differentiation medium induced a much more robust adipogenesis in these cells. Consistent with the insulin-resistant nature of Akt signaling in the *Tsc2^−/−^* MEFs, removal of insulin from the differentiation mixture only mildly reduced intracellular triglyceride levels (Figure 2B). These data suggest that a primary role of insulin and IRS protein function in adipogenesis is to stimulate the downstream activation mTORC1.

Due to increased expression of the insulin receptor (InR) and IRS proteins, the responsiveness to insulin generally increases upon adipocyte differentiation [63], [64]. Therefore, we examined the response of *Tsc2^+/+^* and *Tsc2^−/−^* adipocytes to overnight full serum or acute insulin. As in undifferentiated MEFs (Figure S1), Akt phosphorylation is unresponsive to both serum and insulin in Tsc2-deficient adipocytes, even at the high dose of insulin found in the differentiation mixture, and these cells contain very low IRS-1 protein levels (Figure 2C). While the transcripts for *Inr*, *Irs-1*, and *Irs-2* are all induced in *Tsc2^+/+^* and *Tsc2^−/−^* cells during differentiation, their relative expression levels, especially for *Irs-1* and *Irs-2*, were significantly lower in the *Tsc2^−/−^* adipocytes (Figure 2D). Consistent with previous studies demonstrating that chronic mTORC1 activation decreases both the expression and stability of IRS-1 and IRS-2 [53], [54], [62], we find that 24 h treatment with rapamycin increases *Irs-1* and *Irs-2* transcript levels and IRS-1 protein levels in *Tsc2^−/−^* adipocytes, albeit to levels still significantly lower than *Tsc2^+/+^* adipocytes (Figure S3, panels A and B).

GLUT-4 is a glucose transporter whose translocation to the plasma membrane is regulated by insulin-stimulated Akt signaling (reviewed in reference [65]). GLUT-4 is transcriptionally up-regulated during adipocyte differentiation in order to assure that glucose uptake into adipose tissue is tightly coupled to the insulin response. However, GLUT-4 levels have been found to be decreased in the adipose tissue of a variety of rodent models of insulin resistance (e.g., reviewed in reference [66]). Interestingly, unlike other adipocyte markers, *Glut-4* transcript levels are not induced in differentiating *Tsc2^−/−^* cells, with expression levels being approximately five-fold lower in fully differentiated *Tsc2^−/−^* adipocytes than in *Tsc2^+/+^* adipocytes (Figure 2D). The increased lipid levels in *Tsc2*-deficient adipocytes indicate that these cells must possess alternative mechanisms of carbon/glucose uptake. Previous studies have suggested that, along with a decrease in GLUT-4 expression [67], [68], conditions of obesity and insulin resistance can be accompanied by increased expression of the constitutive glucose transporter GLUT-1 in adipose tissue (e.g., reference [69]). The *Glut-1* gene is a target of the hypoxia-inducible factor-1α (HIF1α) transcription factor, which is upregulated by mTORC1 signaling in TSC-deficient cells ([70], [71]; K.D. and B.D.M., unpublished data). Indeed, *Glut-1* transcript levels were found to be elevated in the *Tsc2^−/−^* adipocytes relative to their wild-type counterparts, and rapamycin treatment blocked this expression (Figure 2E). However, rapamycin had only minimal effects on *Glut-4* expression (Figure 2F). These findings demonstrate that *Tsc2*-deficient adipocytes are insulin resistant and mimic some aspects of insulin resistant adipose tissue from rodent models, including down-regulation of IRS proteins and GLUT-4 and increased expression of GLUT-1.

### *Tsc2* knockdown in 3T3-L1 preadipocytes enhances adipogenesis

**Figure 3:**
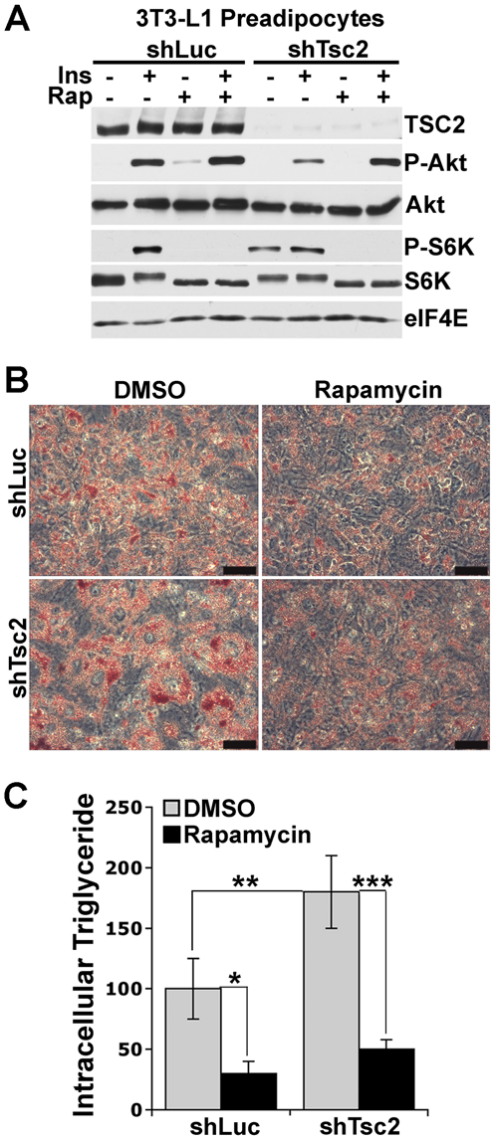
shRNA-mediated knockdown of *Tsc2* expression in 3T3-L1 preadipocytes leads to enhanced mTORC1-dependent adipocyte differentiation. (A) 3T3-L1 preadipocytes with stable shRNA-mediated knockdown of *Tsc2* expression display elevated basal mTORC1 signaling and attenuation of insulin-stimulated Akt phosphorylation. 3T3-L1 preadipocytes stably expressing shRNAs targeting *Tsc2* (shTsc2), or firefly luciferase (shLuc) as a control, were serum-starved for 16 h and, where indicated, were pretreated with rapamycin for 15 min prior to stimulation with 100-nM insulin for 15 min. Lysates were immunoblotted for the indicated phosphorylated (P-Akt (S473), P-S6K1 (T389)) or total proteins with eIF4E levels provided as a loading control. (B) Enhanced mTORC1-dependent adipocyte differentiation of 3T3-L1 preadipocytes following shRNA-mediated knockdown of *Tsc2*. 3T3-L1 preadipocytes expressing shLuc or shTsc2 were induced to differentiate for 9 days in the presence or absence of rapamycin (20 nM), and cells were then stained with Oil Red O. Representative microscopic fields of view (scale bars = 25 µm) are shown at the same magnification for cell line and treatment. (C) Decreased *Tsc2* expression in 3T3-L1 adipocytes results in an mTORC1-dependent increase in intracellular triglyceride levels. 3T3-L1 preadipocytes expressing shLuc or shTsc2 were treated as in (C), and relative triglyceride levels, normalized to cellular protein content, were determined and are shown from three different experiments as the mean±SEM relative to levels in shLuc cells. *P<0.05, **P<0.01, ***P<0.001.

In order to further confirm the role of the TSC1-TSC2 complex and mTORC1 in regulating adipocyte differentiation, we utilized 3T3-L1 preadipocytes as a well-established model for this process. We generated 3T3-L1 cells stably expressing shRNAs targeting *Tsc2* or firefly luciferase as a control. Efficient shRNA-mediated knockdown of *Tsc2* leads to constitutive activation of mTORC1 in 3T3-L1 preadipocytes, as detected by increased basal phosphorylation of its downstream target S6K1 (Figure 3A). Furthermore, these cells exhibit mTORC1-driven insulin resistance, as detected by a decrease in the insulin-stimulated phosphorylation of Akt, which is rescued by pre-treatment with rapamycin. Consistent with the findings in differentiated *Tsc1^−/−^* and *Tsc2^−/−^* MEFs, *Tsc2* knockdown yields adipocytes displaying enhanced oil red O staining (Figure 3B) and increased intracellular triglycerides (Figure 3C) relative to control cells. As expected, rapamycin blocks lipid accumulation in both control and *Tsc2* knockdown cells, further demonstrating an essential role for mTORC1 in adipogenesis.

### mTORC1 drives PPARγ mRNA expression

**Figure 4:**
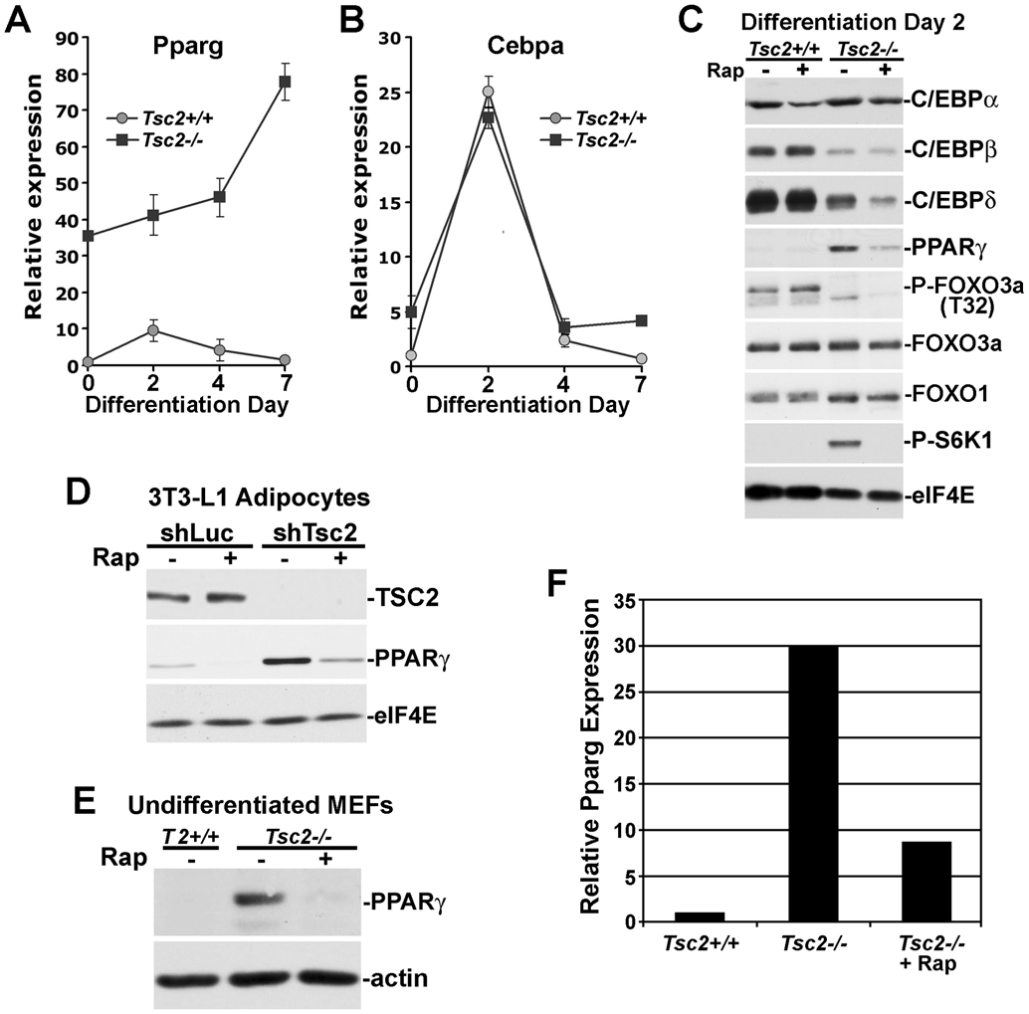
mTORC1 activation triggers a large increase in PPARγ mRNA and protein levels. (A) *Pparg* transcript levels are elevated in *Tsc2^−/−^* MEFs before and during adipocyte differentiation. *Tsc2* ^+/+^ and *Tsc2* ^−/−^ MEFs were induced to differentiate into adipocytes (see Materials and Methods). *Pparg* transcript levels were measured at days 0, 2, 4, and 7 of differentiation by quantitative RT-PCR and are presented as mean±SEM relative to day 0 *Tsc2* ^+/+^ MEFs. (B) *Cebpa* transcript levels are shown in the samples from (A). (C) PPARγ protein levels are increased in differentiating *Tsc2* ^−/−^ cells in an mTORC1-dependent manner. *Tsc2* ^+/+^ and *Tsc2* ^−/−^ MEFs were induced to differentiate for 48 h in the presence or absence of rapamycin (20 nM). Cell lysates were then immunoblotted for levels of phosphorylated (P-S6K1 (T389)) or total proteins, with eIF4E provided as a loading control. (D) PPARγ protein levels are elevated in an mTORC1-dependent manner in *Tsc2*-knockdown 3T3-L1 adipocytes. 3T3-L1 preadipocytes expressing shLuc or shTsc2 were induced to differentiate for 9 days in the presence or absence of rapamycin (20 nM) prior to immunoblotting with the indicated antibodies. (E) Prior to differentiation, PPARγ protein levels are elevated in an mTORC1-dependent manner in *Tsc2^−/−^* MEFs. Where indicated, *Tsc2^−/−^* MEFs were treated for 24 h with rapamcyin (20 nM). (F) *Pparg* transcript levels are elevated in an mTORC1-dependent manner in *Tsc2^−/−^* MEFs. *Pparg* transcript levels were measured in confluent undifferentiated MEFs. Where indicated, *Tsc2^−/−^* MEFs were treated for 24 h with rapamcyin (20 nM). Quantitative RT-PCR results are normalized to the Tsc2^+/+^ control siRNA samples.

In order to explore the mechanism of mTORC1-driven adipogenesis, we focused on components of the well-known transcriptional cascade underlying this differentiation program (reviewed in reference [72]). In differentiation medium, transcript levels for the adipogenic transcription factors PPARγ and C/EBPα peak at day 2 of differentiation in *Tsc2^+/+^* MEFs (Figure 4, panels A and B). *Pparg* transcript levels are strikingly elevated in *Tsc2*-deficient MEFs, even at day 0 of differentiation, and remain high throughout adipogenesis (Figure 4A). However, *Cebpa* transcript levels follow a similar pattern in differentiating *Tsc2^+/+^* and *Tsc2^−/−^* cells (Figure 4B). The protein levels of the major adipogenic transcription factors were examined following 48 h of differentiation in the presence or absence of rapamycin (Figure 4C). Of these factors, only PPARγ levels are elevated in differentiating *Tsc2^−/−^* MEFs relative to *Tsc2^+/+^* MEFs, and in fact, C/EBPβ and δ levels are decreased in these cells. Importantly, the increase in PPARγ levels is sensitive to rapamycin. It is worth noting that the levels of FOXO family members are similar in wild-type and Tsc2 null cells at this stage of differentiation, and consistent with loss of Akt signaling, these transcription factors remain hypophosphorylated in the null cells (Figure 4C). An mTORC1-dependent increase in PPARγ is also seen in the 3T3-L1 adipocytes, described above, following stable shRNA-mediated knockdown of *Tsc2* (Figure 4D). Prior to differentiation, *Tsc2^−/−^* MEFs express greatly elevated levels of both the PPARγ protein (Figure 4E) and transcript (Figure 4F), and these are dependent on elevated mTORC1 signaling, as indicated by their sensitivity to rapamycin. Therefore, loss of the TSC genes results in an mTORC1-driven transcriptional induction of *Pparg*, resulting in elevated levels of PPARγ protein and an enhanced adipogenic program.

### Akt-mediated phosphorylation and inhibition of TSC2 promotes adipogenesis

**Figure 5:**
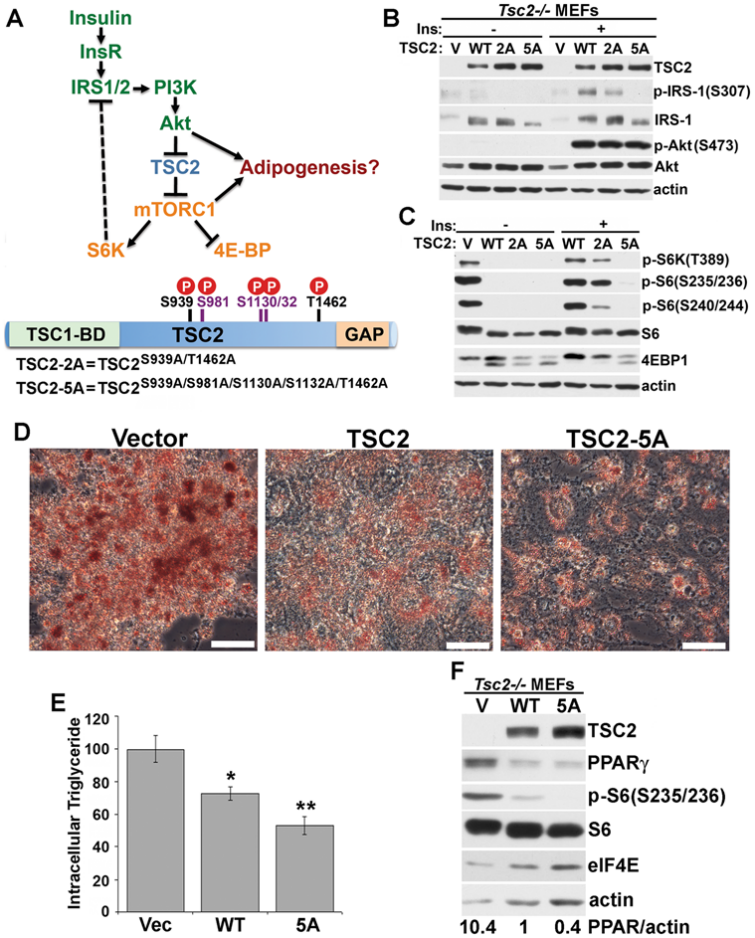
Akt-mediated phosphorylation of TSC2 is essential for insulin-stimulated mTORC1 signaling and is required for proper adipocyte differentiation. (A) Model of insulin stimulation of mTORC1 signaling via Akt-mediated phosphorylation of TSC2 (Top). Akt activation downstream of IRS1/2 and PI3K leads to phosphorylation and inhibition of TSC2, thereby relieving its inhibition of mTORC1 signaling. Consequent activation of mTORC1 leads to inhibition of 4E-BP, activation of S6K, and S6K-mediated feedback inhibition of IRS1/2. Akt phosphorylates TSC2 on five sites (bottom), two of which are conserved in *Drosophila* (depicted in black) and three of which are specific to vertebrate TSC2 (depicted in purple). Alanine substitution mutants affecting the sites conserved in *Drosophila* (TSC2-2A) or all five sites (TSC2-5A) were generated. (B) Reconstitution of *Tsc2* null MEFs with wild-type *TSC2*, *TSC2-2A*, or *TSC2-5A* restores IRS-1 protein levels and insulin responsive Akt phosphorylation. *Tsc2* ^−/−^ MEFs were reconstituted with wild-type TSC2 (WT), TSC2-2A (2A), or TSC2-5A (5A) via retroviral infection. Stable pools were serum-starved overnight and stimulated with insulin (100 nM) for 30 minutes prior to lysis and immunoblotting with the indicated antibodies. (C) Phosphorylation of both the sites conserved in *Drosophila* TSC2 and those specific to vertebrate TSC2 is essential for insulin stimulated mTORC1 signaling. Cells were treated as in (B). (D) The Akt phosphorylation sites on TSC2 are important for adipogenesis. *Tsc2* ^−/−^ MEFs stably reconstituted with wild-type TSC2 (WT), TSC2-2A (2A), or TSC2-5A (5A) were induced to differentiate into adipocytes for seven days and were stained with Oil Red O. Representative microscopic fields of view (scale bars = 25 µm) are shown. (E) Reduced intracellular triglyceride in *Tsc2^−/−^* cells reconstituted with wild-type TSC2 or the TSC2-5A mutant. Relative intracellular triglyceride levels, normalized to cellular protein content, were determined for the cells described in (D) and are shown from three different experiments as the mean±SEM relative to levels in the vector reconstituted cells. *P<0.01 v. vector, **P<0.001 v. vector and *P*<0.05 v. WT. (F) The elevated mTORC1 signaling and PPARγ expression in *Tsc2^−/−^* MEFs is reduced by reconstitution with wild-type TSC2 or the TSC2-5A mutant. The stably reconstituted pools of MEFs described in (B) were lysed at day 0 of differentiation, normalized for total protein, and immunoblotted with the indicated antibodies. Actin and eIF4E levels are provided as loading controls. Relative levels of PPARγ were determined by quantification of band intensity, normalized to actin, using the ImageJ software, and values are expressed relative to the wild-type TSC2-reconstituted cells.

Given the well-defined signaling changes in *Tsc2^−/−^* cells, where constitutive mTORC1 signaling is accompanied by loss of Akt activity, the above data strongly suggest that mTORC1 activation downstream of Akt is sufficient to drive adipogenesis. A primary mechanism by which Akt activates mTORC1 is through phosphorylation and inhibition of TSC2 within the TSC1-TSC2 complex (Figure 5A; [21], [22]). Akt has been found to phosphorylate four or five distinct sites on TSC2, but only two of these sites are conserved and phosphorylated in *Drosophila* TSC2 (S939 and T1462; [73]). Previous studies have found that overexpression of a mutant of TSC2 lacking these two conserved sites (TSC2^S939A/T1462A^, referred to here as TSC2-2A) can dominantly inhibit insulin-stimulated mTORC1 signaling [22]. However, in similar overexpression studies in wild-type cells, roles for the additional phosphorylation sites, which are conserved in TSC2 from all vertebrates, have also been suggested [21], [74]. In order to more rigorously test the relative importance of these phosphorylation sites under conditions where TSC2 is expressed at more physiological levels without interference from endogenous TSC2, we stably reconstituted *Tsc2^−/−^* MEFs with empty vector, wild-type *TSC2*, *TSC2-2A*, or a mutant lacking all five previously identified Akt sites (TSC2^S939A/S981A/S1130A/S1132A/T1462A^, referred to here as *TSC2-5A*) via retrovirus infection. As expected, introduction of wild-type *TSC2* or the phosphorylation-site mutants blocked mTORC1-dependent feedback effects on IRS-1, as detected by decreased S307 phosphorylation, increased electrophoretic mobility, and increased levels of IRS-1 (Figure 5B). Reflecting differential effects on mTORC1 signaling, the TSC2-5A mutant renders a more complete loss of S307 phosphorylation and downshift of the IRS-1 protein than wild-type TSC2 or the TSC2-2A mutant. Acute insulin stimulation of Akt phosphorylation was restored in the three TSC2-reconstituted lines but not in cells expressing empty vector, which remained insulin resistant.

Both wild-type TSC2 and the phosphorylation-site mutants blocked the constitutive, growth factor-independent mTORC1 activity in *Tsc2*-deficient cells, as monitored by S6K1 and S6 phosphoryation and 4E-BP1 mobility shifts indicative of phosphorylation status (Figure 5C). Cells expressing wild-type TSC2 displayed insulin-stimulated mTORC1 signaling, while the TSC2-2A mutant partially attenuated this response. Importantly, mTORC1 signaling was unresponsive to insulin in cells expressing the TSC2-5A mutant, demonstrating that the additional phosphorylation sites on vertebrate TSC2, not found on *Drosophila* TSC2, are essential for Akt-mediated activation of mTORC1.

In order to determine the adipogenic role of TSC2 phosphorylation and inhibition by Akt, we compared the capacity of *Tsc2^−/−^* MEFs expressing empty vector, wild-type TSC2, or the TSC2-5A mutant to differentiate into adipocytes. In support of our findings with littermate-derived wild-type and TSC-deficient MEFs (Figure 1), *Tsc2^−/−^* MEFs reconstituted with wild-type *TSC2* yielded fewer adipocytes with a significant reduction in intracellular triglyceride levels compared to vector control cells (Figure 5, panels D and E). Interestingly, *Tsc2^−/−^* MEFs reconstituted with the *TSC2-5A* mutant were further reduced in their capacity to differentiate into adipocytes (Figure 5D) and accumulated lower levels of intracellular triglycerides than the cells expressing wild-type TSC2 (Figure 5E). Consistent with the mTORC1-dependent increases in PPARγ expression detected in *Tsc2*-deficient MEFs and 3T3-L1 cells (Figure 4), a corresponding decrease in PPARγ levels was observed in the *TSC2* and *TSC2-5A*-reconstituted MEFs at the time of differentiation (Figure 5F). Therefore, despite robust restoration of insulin signaling to Akt in the *TSC2-5A*-reconstitutied cells (Figure 5B), loss of the ability of Akt to phosphorylate TSC2 and activate mTORC1 in these cells leads to decreased adipocyte differentiation. These findings demonstrate that TSC2 phosphorylation is a major contributing factor to the essential role of Akt in adipogenesis.

### Renal angiomyolipoma from TSC patients contain fat and smooth muscle-like cells exhibiting elevated levels of mTORC1 signaling and PPARγ

**Figure 6:**
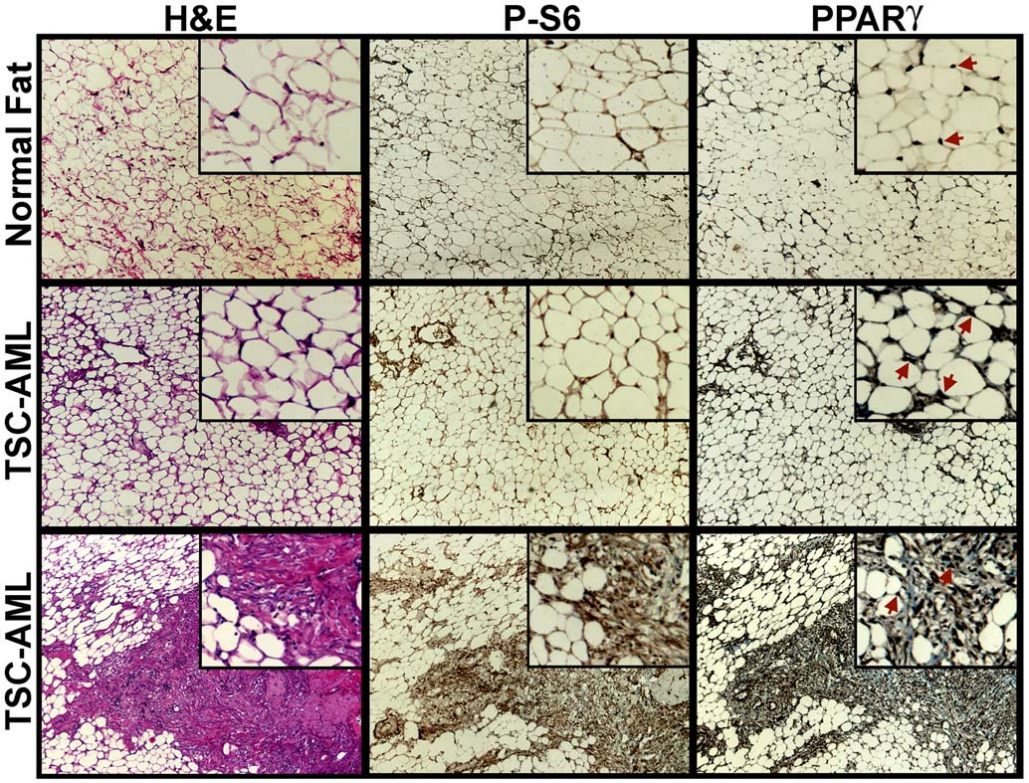
Elevated mTORC1 signaling and PPARγ levels in TSC-associated angiomyolipoma. Serial sections of normal human adipose tissue (top) or renal AMLs (middle and bottom) from TSC patients were H&E stained or subjected to immunohistochemistry with anti-phospho-S6 (S235/S236) or anti-PPARγ, as indicated. Representative regions are shown at 100× magnification, with enlarged (2.5×) insets showing more detail. Red arrows point to a few of the many cells within the field showing nuclear PPARγ.

The mTORC1-dependent adipogenic effects of TSC gene disruption in cell culture, described above, are likely to contribute to the aberrant appearance of adipocytes within renal AMLs from TSC and LAM patients. As with the smooth muscle-like cells within AMLs, these adipoctyes display LOH at the TSC1 or TSC2 locus [49], demonstrating that they are part of the neoplastic lesion. To examine the major signaling defects found in cultured TSC-deficient adipocytes in these tumors, we compared normal human adipose tissue to AML tissue from TSC patients (Figure 6). Phospho-S6 staining was detected predominantly in the cytosol of the adipocytes, which is proximal to the cell surface due to the large lipid droplet, and the AML-associated adipose tissue appeared to have elevated levels of phospho-S6. The smooth muscle-like AML cells also exhibit high levels of phospho-S6 staining (Figure 6, bottom sample). Adipocytes from normal fat tissue contained a single focus of PPARγ staining, indicative of their mononucleated nature. However, the aberrant adipocytes within the AMLs were often multinucleated with each nuclei staining positive for PPARγ (Figure 6, see higher magnification insets). Interestingly, both the adipose and smooth muscle components of the AMLs displayed elevated levels of nuclear PPARγ. In support of these immunohistochemistry findings, data from a gene expression array experiment demonstrate that *PPARG* transcript levels are greatly elevated in human AML samples relative to normal kidney (Figure S4). Therefore, like cell culture models lacking a functional TSC1-TSC2 complex, tumor cells from TSC patients display elevated mTORC1 signaling and PPARγ expression.

## Discussion

While there have been great strides in our understanding of the transcriptional program driving adipocyte differentiation, much less is known regarding upstream signaling pathways instituting hormonal control over the process (reviewed in reference [72]). Amongst critical signaling components downstream of insulin, IRS-1/2 and Akt1/2 have been found previously to be essential for proper upregulation of PPARγ and induction of adipogenesis in cell culture models (e.g., references [1]–[4], [61], [75], [76]). Our study takes advantage of signaling defects triggered by loss of the TSC1-TSC2 complex to determine the contributions of specific branches downstream of these proteins. In this setting where IRS-1 and IRS-2 levels are decreased and Akt is unresponsive to insulin, we find that mTORC1 activation alone can drive PPARγ expression and adipogenesis. As a gain of function model for mTORC1, this study with TSC1/2-deficient MEFs and 3T3-L1 preadipocytes provides critical support to previous studies suggesting involvement of mTOR signaling in PPARγ-driven adipocyte differentiation, which used rapamycin alone to establish this link [12]–[15]. Given that prolonged rapamycin can inhibit mTORC2 assembly and function [45], it is possible that some of the inhibitory effects of rapamycin on differentiation are due to loss of Akt signaling, as detected in at least one study [12]. In cells lacking the TSC1-TSC2 complex, mTORC2 and Akt are already inhibited [55], assuring that the effects of rapamycin on these cells are due to mTORC1 inhibition. Therefore, using rapamycin to inhibit mTORC1 and *Tsc* gene disruption to activate it, our data strongly indicate that mTORC1 activation is both necessary and sufficient to stimulate PPARγ expression and adipocyte differentation.

Our study also identifies TSC2 as a critical downstream substrate of Akt in the induction of adipocyte differentiation. Previous studies have identified five distinct sites on TSC2 that are likely phosphorylated by Akt in vivo [21], [22], [74]. Two of these sites (S939 and T1462) are conserved and phosphorylated in *Drosophila* TSC2 [73], whereas the other three sites (S981, S1130, and S1132) are conserved in all vertebrate versions of TSC2. Previous overexpression studies with phosphorylation-site mutants in otherwise wild-type cells have lead to ambiguous results regarding the relative importance of the different sites [21], [22], [74]. Through the stable reconstitution of *Tsc2^−/−^* MEFs with wild-type and mutant *TSC2*, we find a critical role for the vertebrate-specific sites on TSC2, in addition to those conserved back to *Drosophila*. While the TSC2^S939A/T1462A^ (TSC2-2A) mutant lead to partial attenuation of mTORC1 signaling, expression of the TSC2^S939A/S981A/S1130A/S1132A/T1462A^ (TSC2-5A) mutant resulted in a complete block in the ability of insulin to stimulate mTORC1 signaling events. Importantly, both wild-type TSC2 and the phosphorylation-site mutants were equally efficient at restoring insulin-stimulated Akt signaling to these cells. Therefore, while it is likely that multiple mechanisms exist for Akt to activate mTORC1 signaling, our studies demonstrate that multi-site phosphorylation of TSC2 is essential for this regulation in MEFs. In support of a role for Akt-mediated phosphorylation of TSC2 in promoting adipocyte differentiation, MEFs reconstituted with the TSC2-5A mutant were severely blunted in their ability to take on an adipocyte morphology and accumulate intracellular triglycerides relative to those reconstituted with wild-type TSC2. The fact that some basal differentiation still occurs in these cells, where mTORC1 signaling is blocked, suggests that reactivation of IRS-1/Akt signaling upon TSC2 reconstitution in this setting might provide an mTORC1-independent input into adipocyte differentiation. Therefore, while our study demonstrates a critical role for the Akt-TSC2-mTORC1 signaling pathway in this process, it is likely that other branches of Akt signaling, such as FOXO1 phosphorylation and inhibition [8], play additional and, perhaps, partially redundant roles in promoting adipogenesis.

Our study demonstrates close ties between mTORC1 activation and inhibition and corresponding increases and decreases in both mRNA and protein levels of the adipogenic transcription factor PPARγ, which is both necessary and sufficient for adipocyte differentiation [7]. However, the molecular mechanisms by which signaling pathways regulate PPARγ levels are poorly understood. Previous studies have found that prolonged rapamycin treatment blocks 3T3-L1 adipocyte differentiation, and this coincides with a decrease in both C/EBPα and PPARγ mRNA and protein levels, with no change in C/EBPβ or C/EBPδ [15]. The data presented here demonstrate that mTORC1 activation is sufficient to increase PPARγ expression, even in the absence of other signaling inputs from Akt. We have also examined the mRNA and protein levels of the other known adipogenic transcription factors, which can regulate *Pparg* expression. We have detected an increase in C/EBPα transcript levels in TSC-deficient adipocytes, but unlike PPARγ, we have not detected an increase in C/EBPα protein levels. Furthermore, C/EBPβ and C/EBPδ protein levels are actually lower in differentiating *Tsc2^−/−^* MEFs. Therefore, there appears to be an mTORC1-dependent mechanism promoting *Pparg* transcription.

Renal AMLs are amongst the most prevalent tumor in TSC and LAM patients and are the source of significant morbidity due to their propensity to hemorrhage [47]. In these patient populations, AMLs can grow to sizes greater than the kidney and occupy large areas of the body cavity. The most unusual characteristic of these tumors is that they are comprised of smooth muscle-like cells and adipocytes, both of which display LOH at the *TSC1* or *TSC2* locus [49]. The prevailing model for the origins of this tumor is that a mesenchymal precursor cell undergoes a second genetic hit at one of the TSC loci, rendering the cell defective for TSC1-TSC2 complex function and, thereby, activating mTORC1. Following increased growth and proliferation, the progeny of this cell aberrantly differentiate into these mesenchymal cell types [47]. It seems likely that the mTORC1-dependent increase in PPARγ expression described in this study could account for the abundance of adipocytes in the majority of AMLs from these patients. To this end, we find that both the adipocytes and smooth muscle-like cells in TSC-associated AMLs express high levels of nuclear PPARγ. The somewhat surprising expression of PPARγ in the smooth muscle-like cells suggests that either this transcription factor is also involved in the differentiation of this cell type or that, perhaps, the adipose component of the AML is derived from the smooth muscle through a PPARγ-driven transdifferentiation process. With respect to therapeutics, our findings suggest that PPARγ agonists, such as the thiazolidinedione rosiglitazone, might futher stimulate the putative TSC-deficient precursor cells to differentiate and, therefore, limit their capacity to proliferate and/or migrate.

This study adds adipogenesis to the increasingly diverse array of processes to which mTORC1 activity has been attributed. It is clear that the consequences of mTORC1 activation vary significantly in different cell lineages. Therefore, understanding the role of mTORC1 signaling in human development, physiology, and disease requires studies geared toward defining these tissue-specific functions. Such studies are also important to comprehend the systemic effects of mTORC1 inhibitors, which are now being tested as therapeutics for a number of clinical conditions, including TSC and LAM.

## Supporting Information

Text S1 Supplemental Materials and Methods
(0.03 MB DOC)

Table S1 Quantitative RT-PCR primers used in this study.
(0.04 MB DOC)

Figure S1 MEFs lacking the TSC1-TSC2 complex are defective in Akt signaling. (A, B) Constitutive mTORC1 signaling and insulin-resistant Akt phosphorylation in Tsc1−/− (A) and Tsc2−/− (B) MEFs. Littermate-derived MEFs were serum starved for 16 h and then stimulated with 100- or 830-nM insulin for 15 min prior to lysis and immunoblotting with the indicated antibodies (P-Akt-S473; P-S6-S235/236). (C) Akt-mediated phosphorylation of FOXO1 is defective in Tsc2−/− MEFs. Littermate-derived MEFs were serum starved or grown in full serum overnight prior to lysis and immunoblotting with the indicated antibodies (P-FOXO1-T24). (D) Constitutive localization of FOXO3a to the nucleus in Tsc2−/− cells. GFP-FOXO3a or the Akt phosphorylation site mutant, GFP-FOXO3a-AAA, were transfected into Tsc2+/+ or Tsc2−/− MEFs. GFP fluorescence was then localized in live cells grown in full serum. Representative localization patterns are shown.
(6.57 MB TIF)

Figure S2 Tsc1-deficient MEFs display an mTORC1-dependent increase in adipogenesis. (A) Greater lipid accumulation following an adipocyte differentiation protocol in Tsc1−/− MEFs relative to littermate-derived Tsc1+/+ MEFs. MEFs of the indicated genotype were induced to differentiate for 8 days, and cells were then stained with Oil Red O. Representative culture dish wells (top) and microscopic fields of view (bottom; scale bars = 25 µm) are shown at the same magnification for each genotype. (B) Tsc1−/− adipocytes have higher levels of intracellular triglyceride than Tsc1+/+ adipocytes. MEFs were induced to differentiate for 8 days in the presence or absence of rapamycin (20 nM). Relative triglyceride levels, normalized to cellular protein content, are shown from three different experiments as the mean±SEM. *P<0.01.
(4.11 MB TIF)

Figure S3 Rapamycin increases Irs1 and Irs2 transcript levels in Tsc2−/− adipocytes and partially restores IRS-1 protein levels. (A) Tsc2−/− adipocytes were treated for 24 h with rapamycin (20 nM), and Irs1 and Irs2 mRNA levels were measured by quantitative RT-PCR. Values are normalized to untreated controls and are presented as mean±SEM. (B) Tsc2+/+ and Tsc2−/− adipocytes were treated as in (A) and immunoblotted for IRS-1 protein levels. Total eIF4E levels are provided as a loading control.
(2.14 MB TIF)

Figure S4 PPARG mRNA expression is elevated in kidney angiomyolipomas from TSC patients. Normalized PPARG expression levels in AMLs from two individual patients with TSC are shown relative to expression levels in normal kidney.
(3.58 MB TIF)
