# Supplementary material for: Insulin Stimulates Adipogenesis through the Akt-TSC2-mTORC1 Pathway
Source: PLoS One. 2009 Jul 10;4(7):e6189. doi: 10.1371/journal.pone.0006189 (PMC2703782; doi:10.1371/journal.pone.0006189)
Supplement: Text S1 — Supplemental Materials and Methods (0.03 MB DOC) [file pone.0006189.s001.doc]

**Supplemental Materials and Methods**

**GFP-FOXO3a**

The pcDNA3-GFP-FKHR (GFP-FOXO3a) and pcDNA3-GFP-FKHR;AAA (GFP-FOXO3a-AAA) constructs were kindly provided by the laboratory of W.R. Sellers (Dana-Farber Cancer Center, Boston, MA) and were described previously [77]. These were introduced into MEFs via transient transfection with Lipofectamine 2000 (Invitrogen), according to the manufacturer’s instructions. GFP constructs were imaged using a Zeiss Axio Observer Fluorescence Microscopy System.

**Gene expression analysis in human TSC-associated angiomyolipoma**

Frozen AML tissue from 2 individual patients with TSC (AML548, AML576) were procured from the Maryland Brain and Tissue Bank ([www.btbank.org](http://www.btbank.org/)) in accordance with IRB approved protocols. Total RNA from tumors was harvested using the Trizol Reagent (Life Technologies), while total RNA from pooled-donor normal kidney was obtained from Invitrogen. One microgram of total RNA from each sample was converted to fragmented cRNA according to the Affymetrix protocol and was then hybridized to the Affymetrix GeneChip U133 plus 2.0 oligonucleotide array containing over 54,675 probesets representing more than 38,500 human genes. Hybridized arrays were scanned with the Affymetrix GeneChip® Scanner 3000 and quality control check and data analysis were carried out using Affymetrix GeneChip Operating Software 1.3 and RNA Quality Reporter software (Icoria, Inc.). *PPARG* expression was normalized to the mean expression of the alpha, beta, and gamma isoforms of actin.
