# Supplementary material for: Insulin Stimulates Adipogenesis through the Akt-TSC2-mTORC1 Pathway
Source: PLoS One. 2009 Jul 10;4(7):e6189. doi: 10.1371/journal.pone.0006189 (PMC2703782; doi:10.1371/journal.pone.0006189)
Supplement: Table S1 — Quantitative RT-PCR primers used in this study. (0.04 MB DOC) [file pone.0006189.s002.doc]

Table S1. Quantitative RT-PCR primers used in this study.

| **Mouse Gene** | **Primers (F=Forward; R=Reverse)** |
| --- | --- |
| β-actin | F: gctcttttccagccttcctt  R: cttctgcatcctgtcagcaa |
| Adiponectin | F: ggaacttgtgcaggttggat  R: cccttcagctcctgtcattc |
| Cebpa | F: tggacaagaacagcaacgag  R: tcactggtcaactccagcac |
| Glut1 | F: gctgtgcttatgggcttctc  R: agaggccacaagtctgcatt |
| Glut4 | F: gattctgctgcccttctgtc  R: cagctcagctagtgcgtcag |
| InR | F: ttgaggtgggaaccctactg  R: catcctgcccatcaaactct |
| Irs1 | F: gccagaggatcgtcaatagc  R: ctttcttgttgcttggcaca |
| Irs2 | F: gagccttcagtagccacagg  R: tcaggggtctatccatgctc |
| Leptin | F: ctatgccaccttggtcacct  R: accaaaccaagcatttttgc |
| M36b4 | F: agatgcagcagatccgcat  R: gttcttgcccatcagcacc |
| Pparg | F: cataaagtccttcccgctga  R: gaaactggcacccttgaaaa |
