# Supplementary figures and images for: Insulin Stimulates Adipogenesis through the Akt-TSC2-mTORC1 Pathway
Source: PLoS One. 2009 Jul 10;4(7):e6189. doi: 10.1371/journal.pone.0006189 (PMC2703782; doi:10.1371/journal.pone.0006189)

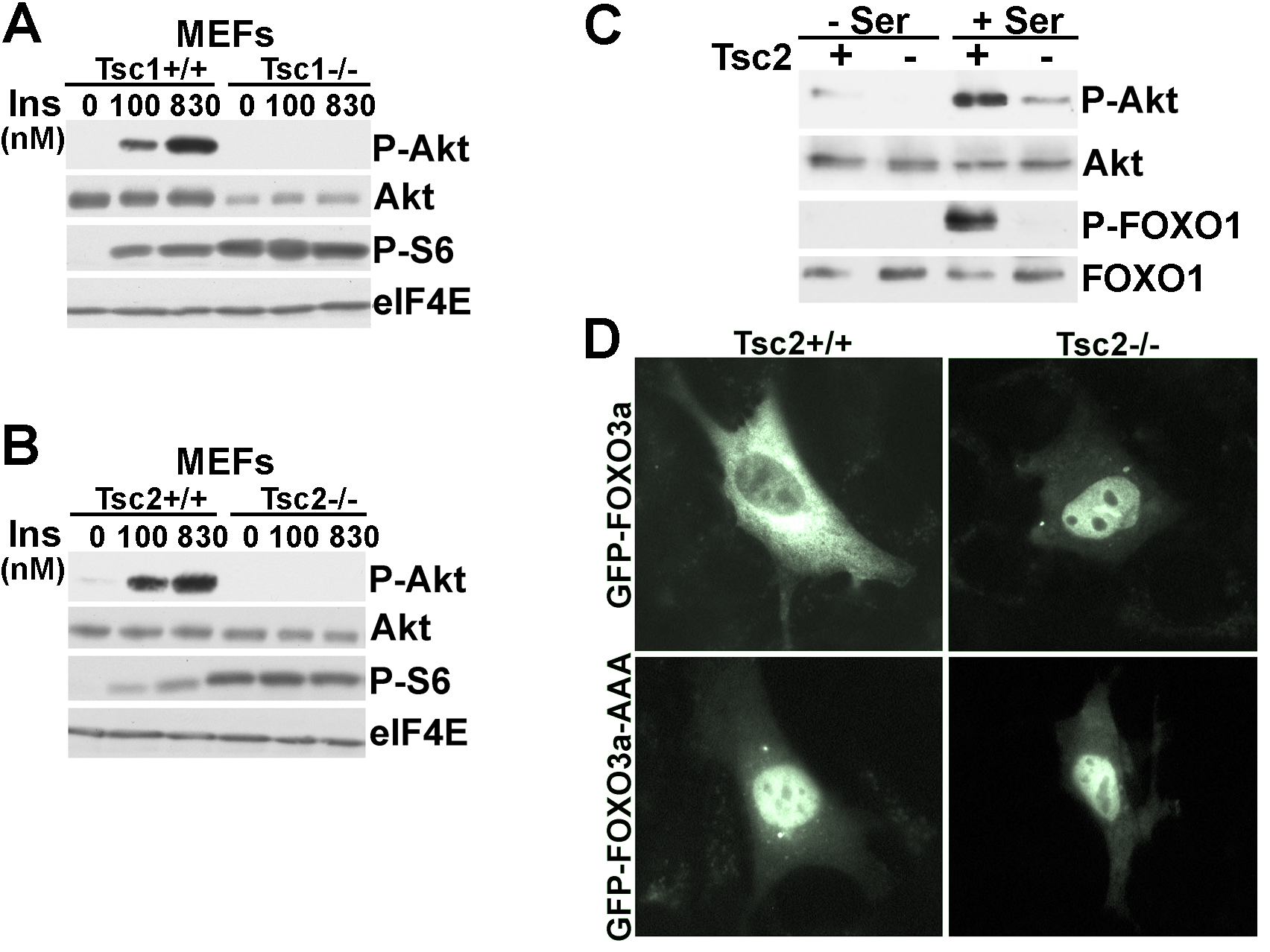

Supplement: Figure S1 — MEFs lacking the TSC1-TSC2 complex are defective in Akt signaling. (A, B) Constitutive mTORC1 signaling and insulin-resistant Akt phosphorylation in Tsc1−/− (A) and Tsc2−/− (B) MEFs. Littermate-derived MEFs were serum starved for 16 h and then stimulated with 100- or 830-nM insulin for 15 min prior to lysis and immunoblotting with the indicated antibodies (P-Akt-S473; P-S6-S235/236). (C) Akt-mediated phosphorylation of FOXO1 is defective in Tsc2−/− MEFs. Littermate-derived MEFs were serum starved or grown in full serum overnight prior to lysis and immunoblotting with the indicated antibodies (P-FOXO1-T24). (D) Constitutive localization of FOXO3a to the nucleus in Tsc2−/− cells. GFP-FOXO3a or the Akt phosphorylation site mutant, GFP-FOXO3a-AAA, were transfected into Tsc2+/+ or Tsc2−/− MEFs. GFP fluorescence was then localized in live cells grown in full serum. Representative localization patterns are shown. (6.57 MB TIF) [file pone.0006189.s003.tif]

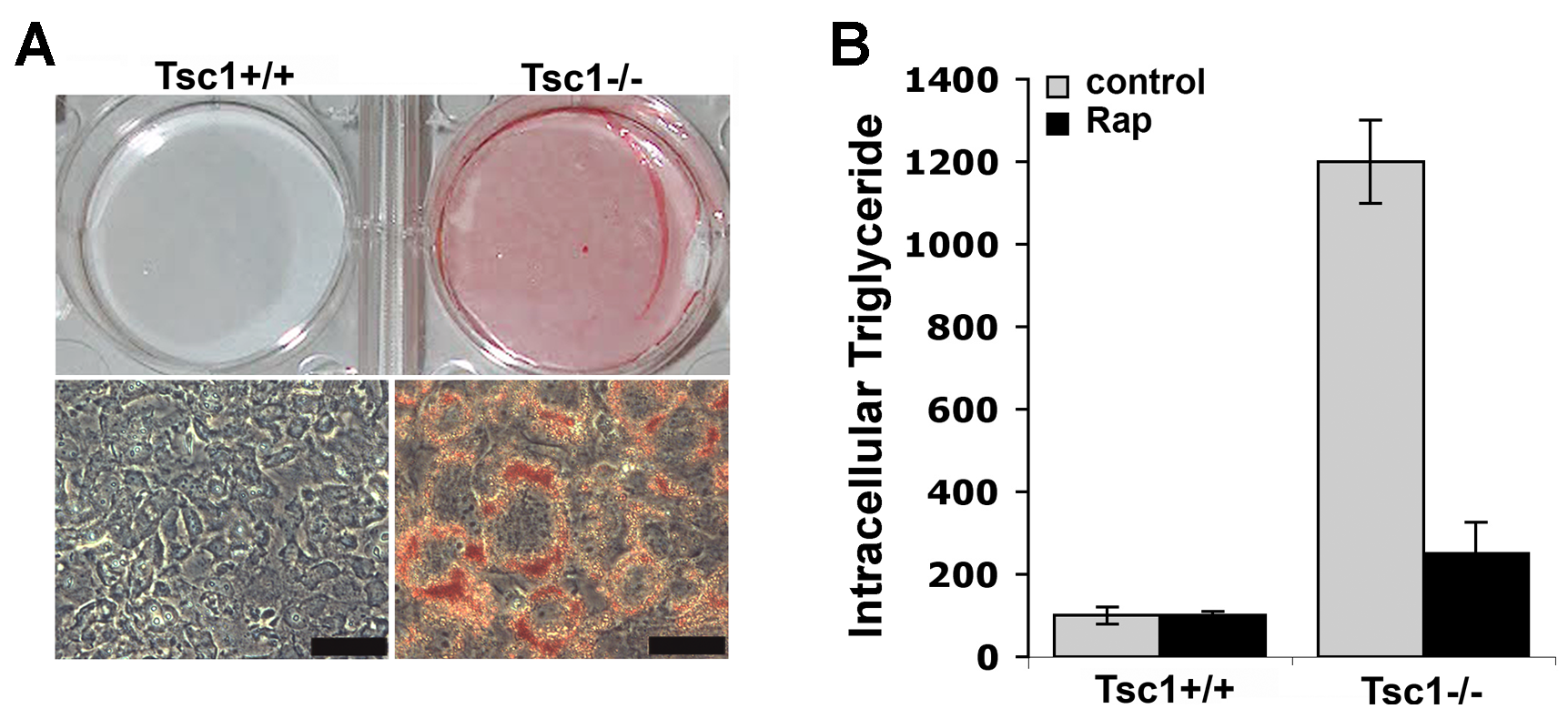

Supplement: Figure S2 — Tsc1-deficient MEFs display an mTORC1-dependent increase in adipogenesis. (A) Greater lipid accumulation following an adipocyte differentiation protocol in Tsc1−/− MEFs relative to littermate-derived Tsc1+/+ MEFs. MEFs of the indicated genotype were induced to differentiate for 8 days, and cells were then stained with Oil Red O. Representative culture dish wells (top) and microscopic fields of view (bottom; scale bars = 25 µm) are shown at the same magnification for each genotype. (B) Tsc1−/− adipocytes have higher levels of intracellular triglyceride than Tsc1+/+ adipocytes. MEFs were induced to differentiate for 8 days in the presence or absence of rapamycin (20 nM). Relative triglyceride levels, normalized to cellular protein content, are shown from three different experiments as the mean±SEM. *P<0.01. (4.11 MB TIF) [file pone.0006189.s004.tif]

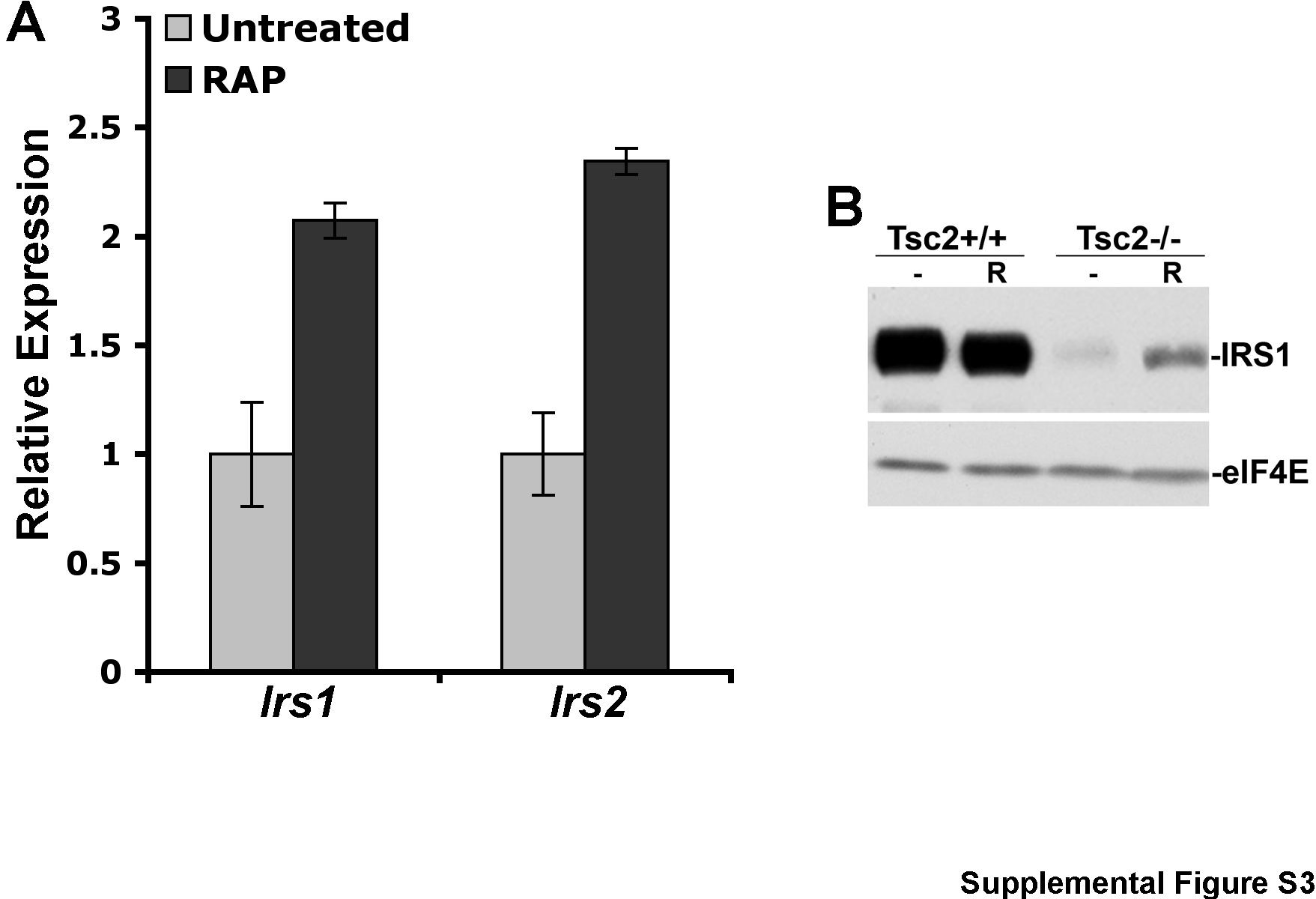

Supplement: Figure S3 — Rapamycin increases Irs1 and Irs2 transcript levels in Tsc2−/− adipocytes and partially restores IRS-1 protein levels. (A) Tsc2−/− adipocytes were treated for 24 h with rapamycin (20 nM), and Irs1 and Irs2 mRNA levels were measured by quantitative RT-PCR. Values are normalized to untreated controls and are presented as mean±SEM. (B) Tsc2+/+ and Tsc2−/− adipocytes were treated as in (A) and immunoblotted for IRS-1 protein levels. Total eIF4E levels are provided as a loading control. (2.14 MB TIF) [file pone.0006189.s005.tif]

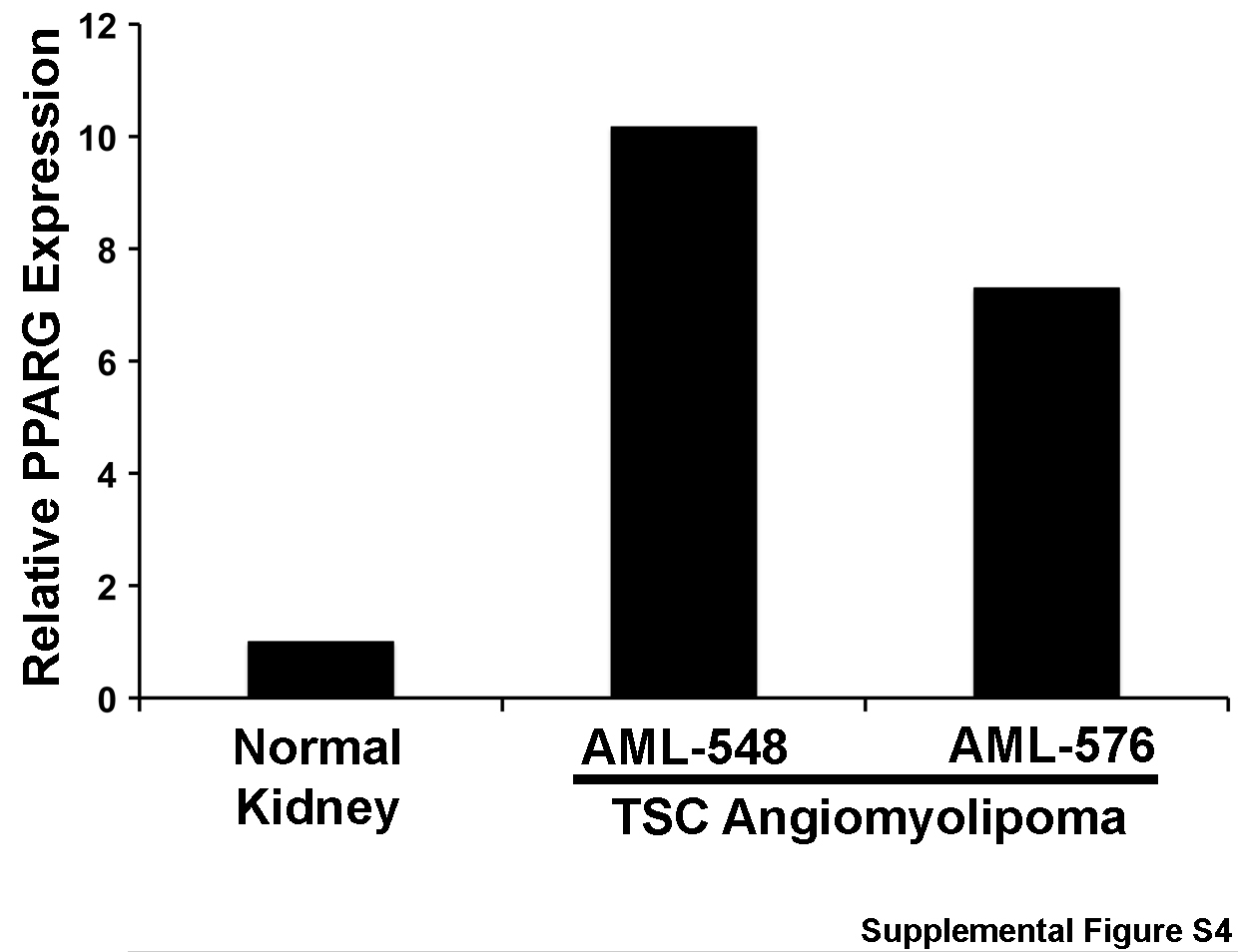

Supplement: Figure S4 — PPARG mRNA expression is elevated in kidney angiomyolipomas from TSC patients. Normalized PPARG expression levels in AMLs from two individual patients with TSC are shown relative to expression levels in normal kidney. (3.58 MB TIF) [file pone.0006189.s006.tif]
